# Design of a Compact Unified SIW Cavity Filtenna Module for Antenna Array Application †

**DOI:** 10.3390/mi16030285

**Published:** 2025-02-28

**Authors:** Andrey Altynnikov, Roman Platonov, Alexey Sosunov, Tatyana Legkova, Andrey Komlev, Andrey B. Kozyrev

**Affiliations:** Department of Physical Electronics and Technology, Saint Petersburg Electrotechnical University “LETI”, ul. Professora Popova 5, 197022 St. Petersburg, Russia; agaltynnikov@etu.ru (A.A.); amsosunov@etu.ru (A.S.); tklegkova@etu.ru (T.L.); aekomlev@etu.ru (A.K.); abkozyrev@etu.ru (A.B.K.)

**Keywords:** filtenna, SIW cavity, bandpass filter, antenna array, slot antenna

## Abstract

The design of a multilayer SIW cavity-fed filtenna is presented. The proposed filtenna can be used as a unified module in an antenna array structure. It consists of three-pole bandpass filter with slot antenna positioned centrally within the top module surface. The modules aperture dimensions of $\lambda_{0}/2\times \lambda_{0}/2$ in conjunction with an SMA feeding port located on the bottom filtenna surface allow implementation of an antenna array of different configurations. This approach allows greatly simplifying the feeding and matching scheme of the array. This module is designed to operate at a 2.655 GHz central frequency with a 70 MHz bandwidth. The procedure of the filtenna design is described in detail. The proposed filtenna was fabricated and tested. The simulation and measurement results show a good agreement. The measurements demonstrate that the maximum measured gain of the prototype is 3.64 dBi with a small variation in the passband.

## 1. Introduction

The ongoing advancement in wireless communication systems raises demands for their key components in terms of their sizes and performance. During evolution, the communications shift from a stand-alone point-to-point link to networks. Each network has its own standards and specifications of frequency bands, channel capacity, etc. To fulfill the demanding criteria of today’s communication standards, devices need to have a smaller size while keeping high performance, low loss, and appropriate power handling capability. However, the variety of different wireless applications leads to a diversity of device designs and technical solutions that should be supported by a single terminal. A clear illustration of this concept can be found in the smartphone industry, where it is essential for one device to support multiple network operations. The ongoing advancement of the IoT is making this challenge more prominent. In general, two possible ways can be marked to overcome this “variety boom”: the unification and functionality convergence of device designs.

The industry tends to prioritize unification, recognizing its potential to deliver an effective trade-off between system performance and the financial investment required for development. However, in multidisciplinary electronics, it may include scientific research as a proving ground, especially for attempting to unite electromagnetic phenomena of different scales [1] or increase functionality integration [2]. The option of functionality convergence can be demonstrated by concepts such as antenna-on-chip (AoC) [3,4], antenna-in-package (AiP) [5,6], active integrated antenna (AiA) [7,8], and filtennas (filtering antennas) [9,10,11]. In contrast to AiP, AoC, etc., the filtenna is a more general concept that can be implemented in different frequency ranges and does not rely on the technological process of device fabrication. The use of the filtenna design allows the miniaturization of the overall size but also improves the system performance by reducing mismatching losses between the filter and antenna. Examples of filtennas in planar design are presented in [9,12,13,14]. Integration of the filtering structure into the feedline is a typical approach for a planar filtenna design. A drawback of these configurations is the requirement to develop complex matching circuits to obtain stable radiation in the frequency range.

Improvement of the passband characteristics can be achieved by the use of cavity resonators in the filtering structure. The substrate integrated waveguide (SIW) is a kind of microwave transmission line that is compatible with PCB manufacturing process. The cavity based on SIW cannot provide quality factors as high as bulk ceramic resonators; however, they can be easily integrated into the feed network of most PCB antennas [10,11,15]. Various methods for constructing antenna designs utilizing SIW technology can be emphasized. One of them is the use of a single layer SIW cavity like the structure presented in [10]. The study utilized integrated electric and magnetic coupling configurations along with fundamental modes to create two distinct radiation nulls. The main disadvantage of this structure is the narrow passband characteristics. In the single layer SIW filtennas, this can be solved by the use of specified topology on the cavity surface. For example, in [15], two U-shaped slots are etched on the cavity surface to provide a radiation null at the left edge of the passband; this allows for an increase in the filtennas’ bandwidth of up to 11.86%.

Another example of using etched slots on the cavity to modify passband characteristics is presented in [11]. This study explores the potential of a single-layer SIW filtenna to operate in dual- or tri-band frequency mode through the implementation of multifunctional cavity-backed slots. Multiple SIW cavity resonators arranged on a single plate can be employed to construct a filtenna design [16,17,18,19,20]. This technique enables a significant improvement in the filtennas’ bandwidth characteristics. In [16], a four-pole cavity SIW filter was designed, while slot antenna were etched on the fourth’s cavity surface. The design proposed in [17] differs by the integration method of a radiation element in the filtenna structure. The patch antenna was considered as a resonator and formed a three-pole filter with two cavity SIW resonators. A significant drawback of using multiple resonator cavities in a single-layer structure is the large dimensions of the device. This is especially critical at low frequencies and when using a high order filter in a filtenna. To overcome this limitation, multilayer SIW structures can be considered [21,22,23,24,25,26]. Researchers presented a filtenna based on a three-pole cavity SIW filter on a three-layer substrate in [23]. The 2 × 2 antenna array using the proposed filtenna structure was designed and fabricated. The filtenna design presented in [22] is a complete analogue of the single-layer filtenna structure with a slot antenna presented in [17]. Coupling between resonators was achieved by using the rectangular slots etched on the metallic layers between them. The slot antenna is integrated into the upper surface of the top cavity resonator. It is worth noting that in [22,23], simple filters without cross coupling between resonators were used. In the work [21], a double slot coupling scheme is used in the middle ground layer between resonators to not only couple them but also to generate two more radiation nulls. This feature significantly improves the out-of-band radiation suppression level of the filtenna. Therefore, they can be used in multiband aperture-shared base station array antenna designs to overcome the mutual coupling of the array elements without increasing the total size of the antenna [27,28,29].

This work is devoted to the design and elaboration of the filtenna module formed by SIW cavities loaded on a radiating slot and based on a multilayer PCB. The distinctive feature of the proposed filtenna design is its modularity and its being “ready-to-use” to assemble array antennas on its base. Thus, the finding of a trade-off between the compactness, universality, and radiation characteristics was the main goal of this research. The area of the proposed filtenna design did not exceed $\lambda_{0}/2\times \lambda_{0}/2$ to provide simple implementation of uniform array antennas. Coaxial PCB compatible connectors (e.g., SMA) were used to feed the filtenna module. The use of connectors allowed simplification of the design of feeding networks for antenna arrays.

This article is a revised and expanded version of a paper entitled “Antenna Module with Integrated SIW Passband Filter for Application in Communication Systems”, which was presented at International Conference on Electrical Engineering and Photonics (EExPolytech), Saint Petersburg, Russian Federation, 18 October 2024 [30]. The specification of the filtenna passband and corresponding design procedure is presented in the following section. Section 3 describes the design of a slot antenna and its integration into the SIW filtering structure. Section 4 presents the fabricated prototype and its frequency and radiating characteristics in comparison with simulated ones. In Section 5, the obtained results are summarized.

## 2. Calculation of Microwave Filter Parameters

**Figure 1:**
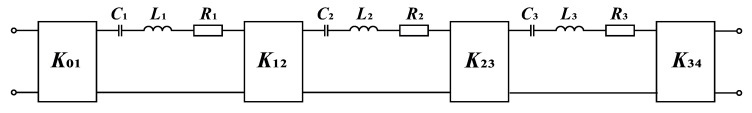
An equivalent circuit of a three-pole bandpass filter.

The initial phase in the filtenna module design involved calculating the parameters of the bandpass filter. The specified parameters for the passband characteristics of the elaborated antenna were as follows: bandwidth (BW) 70 MHz; central frequency 2.655 GHz; return loss (RL) less than −15 dB. The schematic of three-pole filter presented in Figure 1.

Three resonators are presented in the scheme as a serial connection of RLC lumped elements. K-impedance inverters indicate the coupling between adjacent resonators. The design of the passband filter was performed in two steps, following the conventional approach based on the equivalent circuit method [31]. At the first stage, a prototype low-pass filter was calculated. For the Chebyshev three-pole filter, the g-parameters of the low-pass prototype with 0.5 dB ripples in the passband are as follows: $g_{0}$ = 1, $g_{1}$ = 1.5963, $g_{2}$ = 1.0967, $g_{3}$ = 1.5963, $g_{4}$ = 1. The external quality factor of the filter can be calculated using

(1) $$Q_{ext0}=\frac{g_{0}g_{1}f_{0}}{f_{2}-f_{1}}=\frac{g_{3}g_{4}f_{0}}{f_{2}-f_{1}},$$

where $f_{1}$ and $f_{2}$ are the lowest and highest frequency in the passband; $f_{0}$—the central frequency of the passband.

The relations between the K-impedance inverter parameters and the g-parameters of the low-pass prototype filter are as follows:

(2) $$\frac{K_{j,j+1}}{Z_{0}}=\frac{\pi (\lambda_{g1}-\lambda_{g2})}{2\lambda_{g0}\sqrt{g_{j}g_{j+1}}},$$

(3) $$\frac{K_{0,1}}{Z_{0}}=\sqrt{\frac{\pi (\lambda_{g1}-\lambda_{g2})}{2\lambda_{g0}g_{0}g_{1}}},$$

(4) $$\frac{K_{3,4}}{Z_{0}}=\sqrt{\frac{\pi (\lambda_{g1}-\lambda_{g2})}{2\lambda_{g0}g_{3}g_{4}}},$$

where $\lambda_{g0}$—wavelength in a structure corresponds to a central frequency in a bandpass; $\lambda_{g1}$ and $\lambda_{g2}$—wavelength in a structure corresponds to the lowest and highest frequencies in a bandpass, respectively. It was mentioned earlier that the suggested filter configuration is based on a multilayer printed circuit board, with a cavity SIW resonator integrated into each separate layer. The rectangular slots etched in the metallic layer between adjacent resonators are used as coupling elements. These slots may be represented as equivalent series and shunt inductances, with their reactance values calculated in the following way [31]:

(5) $$j\frac{X_{s}}{Z_{0}}=\frac{1-S_{12}+S_{11}}{1-S_{11}+S_{12}},$$

(6) $$j\frac{X_{p}}{Z_{0}}=\frac{2S_{12}}{{\left(1-S_{11}\right)}^{2}-S_{12}^{2}},$$

where $X_{s}$ and $X_{p}$ are the reactances of the slots equivalent series and shunt inductances, respectively; $S_{11}$ and $S_{12}$ are the scattering matrix elements. The relationships between the K-impedance inverter parameters, resonator length $l_{r}$, and both series and shunt inductances of the coupling slots are presented below:

(7) $$\frac{K_{j,j+1}}{Z_{0}}=\left|\tan(-0.5\mathrm{artan}\left(2\frac{X_{p}}{Z_{0}}+\frac{X_{s}}{Z_{0}}\right)+0.5\mathrm{artan}\left(\frac{X_{s}}{Z_{0}}\right))\right|,$$

(8) $$\phi =-\mathrm{artan}\left(2\frac{X_{p}}{Z_{0}}+\frac{X_{s}}{Z_{0}}\right)+0.5\mathrm{artan}\left(\frac{X_{s}}{Z_{0}}\right)-\mathrm{artan}\left(\frac{X_{s}}{Z_{0}}\right),$$

(9) $$l_{r}=\frac{\lambda_{g0}}{2\pi }\left[\pi +0.5(\phi_{r}+\phi_{r+1})\right],$$

The process of the SIW cavity filter design based on a synthesized prototype was organized in the following manner. The parameters of K-impedance inverters were determined by the use of Equations (2)–(4). The model of two SIW cavity resonators coupled by a rectangular slot in a metallic layer in between was formed to provide a full-wave analysis. Metallized dielectric plates with ε=3.5 and tanδ=0.005 were used as the material for a three-layer filter structure. Each layer had an identical thickness of 0.5 mm. The SIW cavity resonator was formed in each dielectric layer. The extent of unwanted radiation emitted by the structure is influenced by the size of the metal holes, denoted as *d*, and the spacing between them, referred to as *p*. To minimize this effect, the value *d* must be less than one fifth of the wavelength in dielectric and more than one half of the *p* value [32]. For determination of the effective cross-sectional width of the SIW resonator for the mode $TE_{101}$ the following expression was used [33]:

(10) $$w_{eff}=w-1.08\left(\frac{d^{2}}{p}\right)+0.1\left(\frac{d^{2}}{w}\right),$$

where *w*—the width of the SIW cavity.

Simulation was performed for the model with different sizes of coupling slots. Once the S-parameters were acquired, Equations (5) and (6) were employed to calculate the reactance of the coupling slots. The determined $X_{s}$ and $X_{p}$ values were used to find the K-impedance inverters’ parameters using (7). Founded values were comprised with results obtained for calculation of K-impedance inverters parameters by employing Equations (2)–(4). The variation in the coupling slot sizes occurred until the results matched.

**Figure 2:**
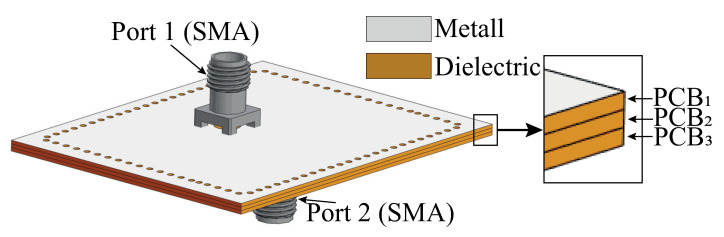
Model of three-pole SIW filter prototype.

**Figure 3:**
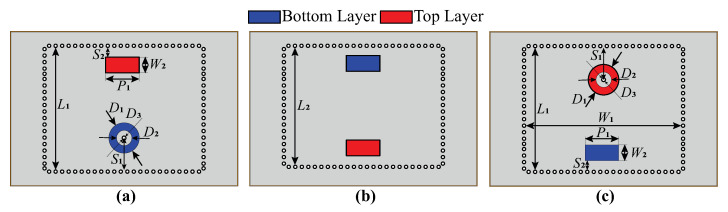
Schematic of filter proyotype layers’ topology: (**a**)—bottom, (**b**)—middle and (**c**)—top substrate.

The filter model and topology of each layer are presented in Figure 2 and Figure 3. The feed point of the SIW cavities was determined with the help of full-wave electromagnetic simulation. The footprint for the coaxial connector is a ring-shaped slot with an external and internal diameter $D_{1}$ = 4.22 mm and $D_{2}$ = 1.27 mm. The SMA connector was used as a terminal, allowing easy prototype performance tests by the use of common coaxial feed lines. The external quality factor can be changed by varying the distance $S_{1}$ between a central pin of the SMA connector and the SIW cavity boundary. The values of geometrical sizes for the filter model are presented in Table 1.

**Figure 4:**
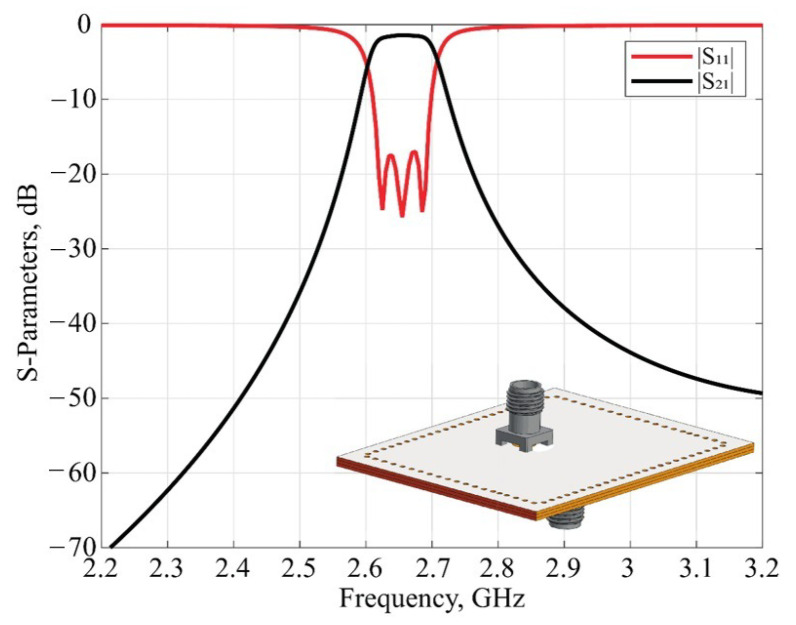
Simulation results of filter prototype.

Figure 4 presents the S-parameters obtained as a result of the designed SIW filter full-wave simulation. One can see that the central frequency of the filter is 2.655 GHz with the bandwidth of 70 MHz. The return loss is more than 18 dB, and the insertion loss is less than 1.5 dB without significant ripples in the passband.

## 3. Design of Filtenna

**Figure 5:**
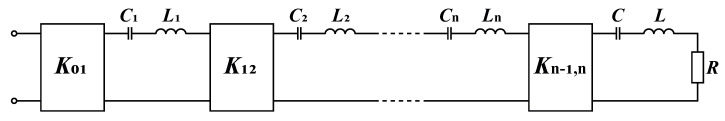
Equivalent circuit of the filtenna based on SIW filter.

The model of the designed SIW bandpass filter was modified to form the filtenna module. For this reason, one of the 50 Ohm terminals was replaced by a radiating slot. The slot antenna was selected as a radiating element for its simplicity of incorporation into the SIW cavity and directional radiation pattern. The equivalent circuit of the slot can be presented by a series connection of *R*, *L*, and *C* elements. The corresponding equivalent circuit of the filtenna is presented in Figure 5. The reactance of the equivalent inductor and capacitor determines the value of resonant frequency $f_{0}$ (corresponding to the central frequency of the passband SIW filter), while the resistance *R* represents effectiveness of the radiation of the radiating slot. These equivalent component values can be calculated using the following equations:

(11) $$L=\frac{1}{4\pi }\frac{d(Im\left(Z_{in}\right))}{df}{|}_{f=f_{0}},$$

(12) $$C=\frac{1}{{\left(2\pi f_{0}\right)}^{2}L},$$

(13) $$R=Z_{in}\left(f_{0}\right),$$

thus, $Q_{ext}$ of the slot can be calculated as

(14) $$Q_{ext}=\frac{2\pi f_{0}L}{R}.$$

**Figure 6:**
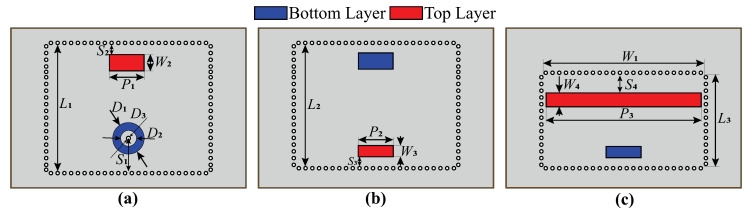
Schematic view of the (**a**)—top, (**b**)—middle and (**c**)—bottom substrate.

It is essential to design a radiating slot so that Equations (1) and (14) provide identical values of the external quality factor. This is required to maintain the frequency response of the designed filtenna. The electromagnetic model of the SIW filter was correspondingly modified for further full-wave analysis and the determination of the optimal slot dimensions. The layout of the SIW cavities for the filtenna module is presented in Figure 6.

**Figure 7:**
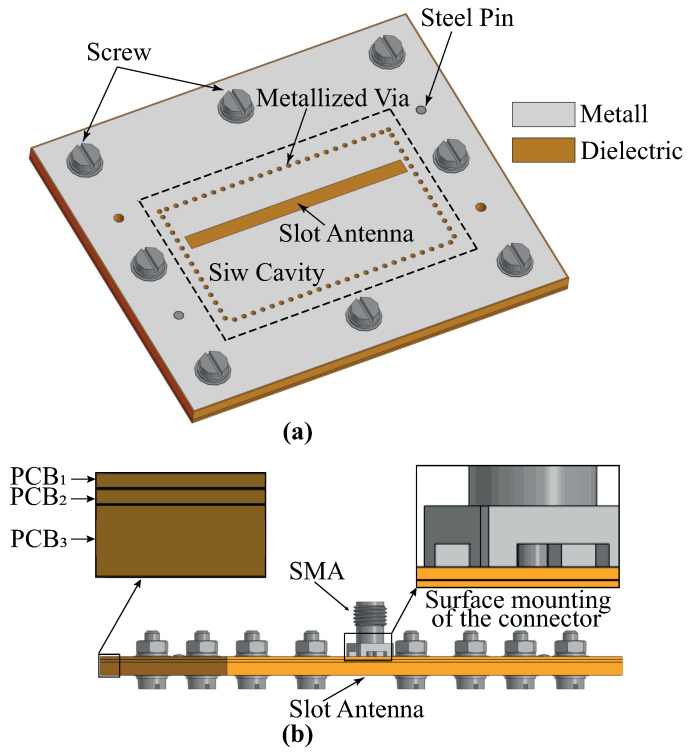
Top view—(**a**) and side view—(**b**) of filtenna model.

During simulation, the optimization of the topology elements was carried out in the time domain [34] using the algorithm of the inverse chirp-z transformation [35]. It was observed that there is no optimal set of parameters $S_{4}$, $W_{4}$, and $P_{3}$, while the thickness of the SIW cavity loaded on the radiating slot is constant. Thus, the value of the loaded cavity thickness was also added to the optimization process. In the end, the filtenna was a three-layer structure with layer thicknesses of 0.5, 0.5, and 2.4 mm. The dimensions of the topology elements of the filtenna’s final design are listed in Table 2. For the assembly of the filtenna prototype, steel pins were used for PCB layer alignment, while the mechanical connection of PCBs was provided by screws. The final design of the filtenna module is presented in Figure 7.

**Figure 8:**
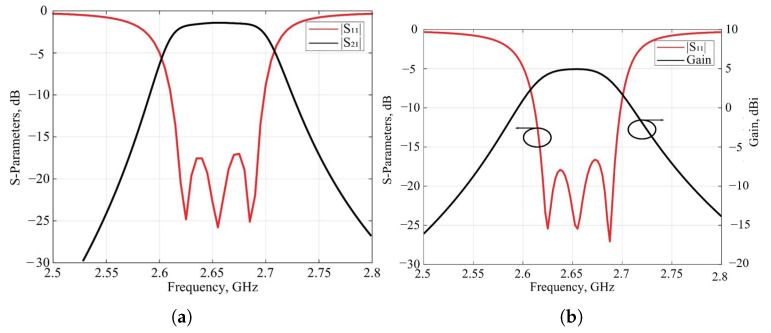
Comparison of the simulation results of the SIW filter prototype—(**a**) and filtenna module—(**b**).

The simulation results of the S-parameters of the SIW filter prototype and filtenna are presented in Figure 8. The integration of the slot antenna into the filter structure, as illustrated in the figure, successfully maintained the filter’s transmission characteristics without any distortion. In Figure 8, the simulated dependence of the filtenna gain on the frequency is presented. The maximal gain value of 4.8 dBi at the central passband frequency is observed. This characteristic shows high uniformity in the frequency passband; the maximal deviation of the antenna gain is 0.4 dBi and can be observed at the edges of the bandwidth only.

**Figure 9:**
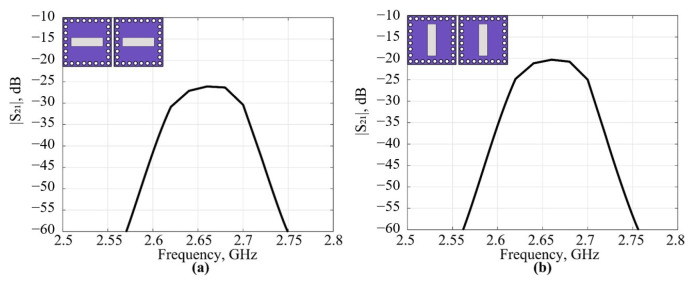
Simulation results of cross coupling of the two filtenna modules oriented in a row— (**a**) and in parallel—(**b**).

The coupling along the apertures of the filtenna modules were simulated to estimate the potential of the filtenna array performance. Two configurations of the filtenna modules were considered (see insets in Figure 9); the distance between the center points of the modules is half of the free-space wavelength at the center frequency of the passband for both configurations. The results show that higher coupling is achieved in the case of the parallel orientation of the radiating slots; however, it is close to −20 dB in the worst case. Thus, the filtenna modules demonstrate good isolation without any additional decoupling technique implemented.

**Figure 10:**
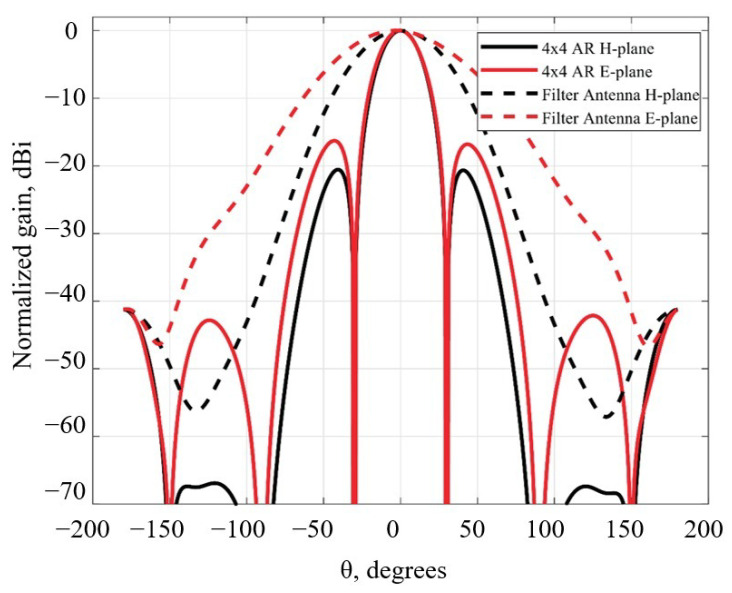
Comparison of the radiation pattern of single module and 16-element array.

The radiation pattern of the 16 filtenna modules was estimated by the array factor (AF) calculation on the basis of the simulated radiation pattern of the single module. The modules were organized as a plane 4 × 4 array with spacing between center points of the modules of half free-space wavelength at the central passband frequency. The comparison between the radiation patterns of the single module and the 4 × 4 array is presented in Figure 10. One can see that the main beam of the radiation pattern for the array has a lower beamwidth (~23 degrees in both planes) in contrast to the single filtenna module (~67 and 50.5 degrees for the E- and H-plane, respectively) in both the E- and H-planes. The sidelobe level less than ~−17 dB was achieved with uniform magnitude distribution among modules and without any suppression technique implemented.

## 4. Experimental Results

The copper-clad composite material based on polytetrafluoroethylene (PTFE) was used as a base material for the PCB fabrication. The weight fraction of TiO_2_ addition of the composite was ~25%. Preliminary samples of composite material were manufactured by hot pressing for experimental investigation of the parameters that were performed by the resonance technique, explained in detail in [36]. The measured dielectric constant value was 3.5±0.07 with a loss tangent about 0.005 at 10 GHz. The layers of the filtenna module were manufactured separately by printed circuit board technology. A layer of immersion silver was applied over the copper metal surfaces to prevent oxidation. The use of screw connections to assemble PCBs into a stack allows eliminating soldering in between. That in turn allows simple the replacing and adjusting of PCB layers. In contrast to other filtenna designs [21,22,23,37,38], the central pin of the SMA connector does not pass through the board; it is soldered to the metallic pad of the coaxial excitation point (see $D_{1}$ and $D_{2}$ in Figure 6). Such an approach allows replacing the SMA connector with any mechanically compatible connector that fits the feed point footprint.

**Figure 11:**
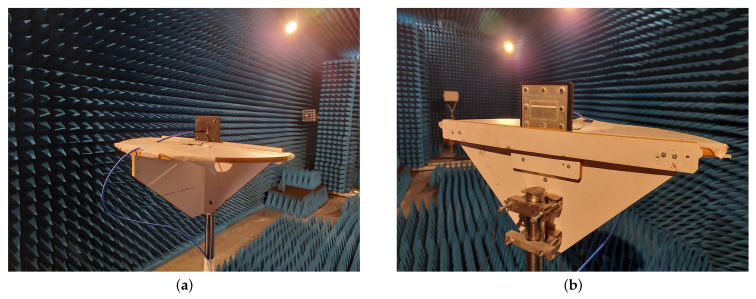
Photos of the assembled filtenna module prototype in the anechoic chamber: (**a**)—back view and (**b**)—front view of the module.

**Figure 12:**
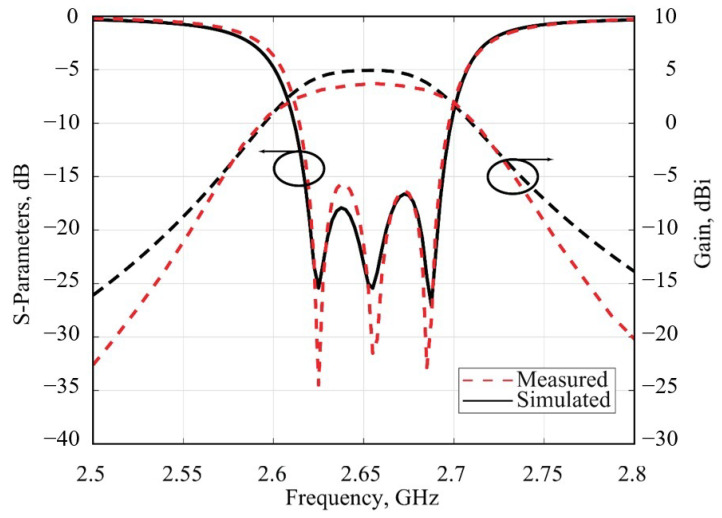
Comparison of the experimental results of the filtenna’s gain and reflection coefficient on the frequency with simulated ones.

**Figure 13:**
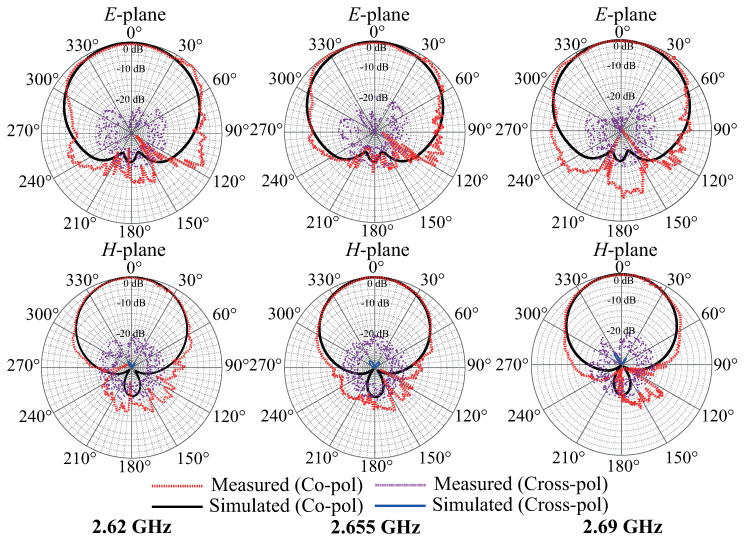
Comparison of the experimental and simulation results of filtenna’s gain at different frequencies.

The experimental investigation of the filtenna’s frequency response and radiation pattern was elaborated with the help of VNA Ceyear 3672C (Ceyear Technologies Co., Ltd. Qingdao, China) in the anechoic chamber environment. The assembled filtenna module during the measurement process is presented in Figure 11. The comparison of the experimental results of the filtenna’s gain and reflection coefficient frequency dependencies with the simulation results is presented in Figure 12. The experimental results show that the designed filter antenna prototype operates at a central frequency of 2.655 GHz with a bandwidth of 70 MHz, which meets the requirements of the Band 7 frequency range used in modern communication systems. The maximum antenna gain is 3.64 dB at the center frequency. Figure 13 presents the measured radiation patterns of the filtenna module at the lower (2.62 GHz)and upper edges (2.69 GHz) of the passband as well as the central frequency (2.655 GHz). The measured level of cross polarization is less than −19 dB in the E-plane and less than −20 dB in the H-plane. It can be seen that the measurement results are in good agreement with the simulation results. The difference between the measured and simulated gain values can be explained through several considerations. Since the filtenna multilayer structure was assembled manually, any small misalignment of the coupling apertures may cause an impedance mismatch that can be seen in Figure 12 near 2.63 GHz. Another reason is the slightly higher value of the loss tangent of the manufactured composite material compared to the simulated one.

The comparison of the proposed filtenna module key parameters with designs published in the last five years is presented in Table 3. One can see that the presented filtenna is the most compact. The high gain values of the considered designs in comparison with the proposed one can be explained by the higher aperture dimensions. Since the gain value depends on the aperture size, a higher area of radiator elements provides better gain. The main drawback of the considered designs, in contrast with the proposed one, is the difficulty of using them as a base element of an antenna array.

## 5. Conclusions

The design of the compact SIW cavity filtenna module was presented. The proposed design demonstrates the possible unification of the filtenna module for application in antenna arrays. The design features simplifying the implementation of the array antenna systems based on proposed modules are the stacked arrangement of the cavities, the slot radiating element positioned centrally on the top module surface, the module aperture dimensions ($\lambda_{0}/2\times \lambda_{0}/2$), and the SMA-compatible feeding port located on the bottom filtenna surface. The synthesis technique of the proposed filtering antenna module was described. A filtenna module operating in the LTE Band 7 at a center frequency of 2.655 GHz with a bandwidth of 70 MHz was designed and fabricated. The PTFE-based composite material was manufactured and used for the filtenna’s PCB fabrication. The measured permittivity of the composite material was close to 3.5, with a loss tangent value of about 0.005. The simulation and measurement results were in good agreement. The gain value dependence on the frequency for the fabricated module showed high uniformity in the frequency passband with a maximum value of 3.64 dBi. The measured cross-polarization level was below −19 dB/−20 dB in the E/H-plane. The proposed compact filtenna offers the advantages of low cost and simple configuration with stable in-band radiation performance characteristics and excellent out-of-band selectivity. These advantages empower it suitably for practical applications in many wireless mobile space-limited platforms.

## Figures and Tables

**Table 1:** Dimensions of filter prototype elements.

$L_{1}$ mm	$L_{2}$ mm	$S_{1}$ mm	$S_{2}$ mm	$W_{1}$ mm	$W_{2}$ mm	$P_{1}$ mm	$D_{1}$ mm	$D_{2}$ mm	$D_{3}$ mm
38.15	36.84	4.5	2	47.8	3	8.96	4.22	1.27	0.5

**Table 2:** Dimensions of filtenna elements.

$L_{1}$ **mm**	$L_{2}$ **mm**	$L_{3}$ **mm**	$S_{1}$ **mm**	$S_{2}$ **mm**	$S_{3}$ **mm**	$S_{4}$ **mm**	$P_{1}$ **mm**	$P_{2}$ **mm**
38.84	37.03	30.21	14.7	2	2	6.48	6.4	6.31
**$P_{3}$ mm**	**$D_{1}$ mm**	**$D_{2}$ mm**	**$D_{3}$ mm**	**$W_{1}$ mm**	**$W_{2}$ mm**	**$W_{3}$ mm**	**$W_{4}$ mm**	
46	4	2.1	0.5	48.8	3	2	3	

**Table 3:** Comparison with reported SIW filtennas.

Ref. No	Volume × $10^{3}$, $\lambda_{0}^{3}$	Central Frequency, GHz	Bandwidth, %	Gain, dB	Radiator Type	Feed Line
[18]	47.41	~4.4	5.4	6.5	Slot	Coaxial
[20]	20	3.5	9.14	~7	Slot	Microstrip
[26]	14.25	5.365	7.64	5.3	Slot	Microstrip
[24]	5.33 | 15.23	2.822 | 4.006	2.1 | 4.1	4.9 | 5.38	Slot	Microstrip
[25]	26 | 39	3.59 | 4.11	2.3 | 1.6	4.84 | 4.85	Slot	Coaxial
[28]	5	3.64	4.29	3.66	Patch	Microstrip
[39]	15.36	4.88	9	7.7	Patch	Coaxial
[19]	18.8	4.8	8.3	10	Slot	Coaxial
This work	4.5	2.655	2.64	3.64	Slot	Coaxial

## Data Availability

The original contributions presented in the study are included in the article; further inquiries can be directed to the corresponding author.
